# Detection of *K. pneumoniae* Hospital-Acquired Strains That Produce Carbapenemases in Thrace Tertiary Hospital

**DOI:** 10.3390/microorganisms13112496

**Published:** 2025-10-30

**Authors:** Anastasia Vezyridou, Aikaterini Skeva, Ioanna Alexandropoulou, Valeria Iliadi, Georgios Euthymiou, Dimitrios Themelidis, Athina Xanthopoulou, Vasilios Petrakis, Theocharis Konstantinidis, Maria Panopoulou

**Affiliations:** 1 Laboratory of Microbiology, Department of Medicine, Democritus University of Thrace, Dragana, 69100 Alexandroupolis, Greece; anasvezy95@gmail.com (A.V.); skevakaterina@hotmail.com (A.S.); ialexand.mbg@gmail.com (I.A.); iliadi.valeria18@gmail.com (V.I.); euthymiougiorgos@gmail.com (G.E.); dthemelidis@gmail.com (D.T.); athina.xanthopulu@gmail.com (A.X.); mpanopou@med.duth.gr (M.P.); 2 Second Department of Internal Medicine, University General Hospital Alexandroupolis, Democritus University of Thrace, 68100 Alexandroupolis, Greece; vasilispetrakis1994@gmail.com

**Keywords:** *Klebsiella pneumoniae*, carbapenemases, klebsiella producing carbapenemase (KPC), metallo-β-lactamases (MBLs)

## Abstract

In recent decades, the problem of resistant strains, which present resistance to different types of antimicrobials, has increased. *Klebsiella pneumoniae* is one of the most important species that exhibits an acquired resistance phenotype to at least one agent in three or more classes of antimicrobials and is thus characterized as a multidrug-resistant bacterium (MDR). 98 nosocomial strains of *K. pneumoniae* were isolated during the pre-COVID-19 period, and more specifically, from February 2015 to March 2019, were analyzed for the detection of class A, D, and B carbapenemase genes. The existence of KPC, OXA-48 like, IMP, VIM, and NDM carbapenemases has been examined. The immunochromatography showed that NDM carbapenemases are more frequently detected in the samples, reaching a percentage of 30.7%, while correspondingly the percentage for VIM carbapenemases was 7.68% among the strains with resistant phenotypes. No strain with carbapenemase IMP was found. Real-time multiplex polymerase chain reaction (PCR) showed, in contrast to immunochromatography kits, that a high percentage of bacterial isolates (94.26%) carry NDM and VIM carbapenemase genes, while no IMP carbapenemase genes were detected. Regarding the KPC enzymes, the immunochromatography kits showed that KPC positive strains are reaching 53.1%, and OXA-48 positive strains are reaching 3.1% among the strains with resistant phenotypes. Real-time multiplex polymerase chain reaction revealed a much higher percentage of 89.6% KPC positive isolates and a percentage of 14.6% OXA-48 carbapenemase producers. The aforementioned results indicate the dominance of the Multiplex Real-Time PCR as a “gold standard” method. This study could not fully support the usefulness of rapid immunochromatographic tests as a fast and useful diagnostic tool in the laboratory daily routine, as per the results of previous studies. Thus, more studies need to be conducted in this field to introduce these rapid tests safely into the daily laboratory workflow as a screening tool. Additionally, this study underlines the predominance of KPC enzymes from clinical isolates of ICUs and a significant shift over the OXA-48 like enzymes that are not limited to the ICU environment.

## 1. Introduction

Antimicrobial resistance has been identified as a major issue that poses a threat to public health [1,2,3]. In particular, the development of microbial resistance to carbapenems, the last-line antibiotics in the treatment armamentarium of clinicians, by Gram-negative bacilli of the Enterobacteriaceae, especially the studied species *K. pneumoniae*, is a particularly worrisome issue [4,5]. *K. pneumoniae* is a worldwide nosocomial pathogen [6]. Therefore, the emergence of resistant strains of this species against carbapenems in the hospital environment leads to an increasing limitation of available treatment options, with the reintroduction to daily clinical practice of previously used antimicrobials that had been withdrawn, for example, due to toxicity issues [7,8,9,10,11,12,13].

In addition, the appearance of such resistant strains of this species has as a consequence the extension of the hospitalization time of the patients with the corresponding financial consequences through the increasing burden of the health care system, but also considerable are consequences for the health of the patients, increasing the suffering and possible complications due to infections caused by such strains of the species [14].

An important mechanism for the development of microbial resistance is the ability to produce β-lactamase enzymes and, in particular, carbapenemases from carbapenem-resistant strains of the species [15]. At this point it should be emphasized that the phenomenon of microbial resistance to carbapenems in this species is not solely the result of the production of these enzymes, but there may be other coefficients of resistance that contribute to this resistant phenotype. However, these plasmid-encoded carbapenemases have their special place regarding microbial resistance to carbapenems [16].

The phenomenon of antimicrobial resistance has been the epicenter of a significant number of studies due to the need for detection and reporting of such strains of *K. pneumoniae* species capable of producing carbapenemases in the context of epidemiological control and prevention of their spread by the prudent use of carbapenems in medical practice and the observance of necessary measures in the hospital environment settings to limit the potential dispersion of such strains [17,18].

The molecular method revealed a higher percentage of positive results for three enzymes of bacterial strains, supporting its predominance as the reference method for the detection of these enzymes in bacterial strains of the species. In addition, the sensitivity and specificity of the immunochromatographic method (ICT) using the molecular method as a “gold standard” technique did not reach the high percentages shown by other studies in the international literature, which used multiplex rapid detection immunoassays (ICT-FLI) [19,20,21], emphasizing the need for further studies to clarify the significance of these methods as a diagnostic tool in the laboratory routine practice.

In this paper, we aimed to test the ability of *K. pneumoniae* hospital-acquired strains to produce carbapenemases type A (KPC), D (OXA-48 like), and B (VIM, NDM, and IMP), which contribute to the phenomenon of antimicrobial resistance, using two phenotypic methods (an automated Vitek 2 method for the determination of antimicrobial resistance and an immunochromatographic method-ICT), as well as a molecular method (Multiplex Real-Time PCR), which was the reference method of the study for the detection of carbapenemases.

## 2. Materials and Methods

### 2.1. Bacterial Strains

This study includes 98 *Klebsiella pneumoniae* clinical strains, which were isolated from the departments of the University General Hospital of Alexandroupolis from February 2015 to March 2019.

### 2.2. Antibiotic Susceptibility Tests

To achieve the determination of microbial resistance by measuring the minimum inhibitory concentration (MICs) for carbapenem antibiotics (imipenem, meropenem, ertapenem, doripenem), the automated system VITEK-2 systems (bioMerieux, Marcy l’Etoile, France) was used according to the procedures and protocol followed in the daily microbiological workflow to determine the resistance of clinical strains.

At this point, it should be mentioned that, regarding the screening of strains capable of producing carbapenemases, the cut-off of meropenem > 0.125 mg/L (zone diameter < 28 mm) was used, as proposed by the European Commission for Testing of Microbial Susceptibility (EUCAST) (EUCAST Clinical Breakpoint Tables v. 12.0) [22].

### 2.3. Lateral Flow Immunoassay (LFI)—Immunochromatographic Test (ICT)

In order to study the ability of the bacterial strains of *K. pneumoniae* to produce KPC OXA-48 like, VIM, NDM and IMP carbapenemases, the phenotypic method of the Lateral Flow Immunoassay (LFI) was used [23]. Bacterial isolates were spread onto the surface of MacConkey Agar and incubated for 18 to 24 h. Two commercially available kits, NG Test^®^ CARBA 5 (Guipry, France) and RESIST-5 O.O.K.N.V. (Coris BioConcept, Gembloux, Belgium) were used according to the manufacturer’s instructions.

### 2.4. Molecular Methods

#### 2.4.1. DNA Extraction

Total DNA was extracted from *Klebsiella pneumoniae* isolates overnight culture using the commercially available DNA, RNA, and protein purification genomic material extraction kit, MACHEREY-NAGEL (Düren, Germany) Certified Quality, NucleoSpin^®^ Tissue (50 preps), following the manufacturer’s instructions.

#### 2.4.2. Multiplex Real-Time PCR

To screen the 98 bacterial strains for the molecular detection of *bla*KPC, *bla*OXA-48 similar, *bla*VIM, *bla*IMP, and *bla*NDM carbapenemase genes, Multiplex Real-Time Polymerase Chain Reaction (PCR) was performed using two commercially available kits, the Multiplex Real-Time PCR kit MDR KPC/OXA Real-TM Sacace Biotechnologies (Como, Italy) and the Multiplex Real-Time PCR kit MDR MBL VIM/IMP/NDM Real-TM Sacace Biotechnologies (Como, Italy), with some modifications. These modifications are related to the fact that the negative control of extraction (NCE) was not included, and the internal control was also not used (internal control). Nevertheless, the experimental procedure was followed according to the manufacturer’s manual.

### 2.5. Statistical Analysis

The counting data was expressed by rate (%). The independent sample *t*-test was used. Statistical analysis was performed using IBM SPSS 26. All data are presented as mean ± standard deviation (SD). Statistical significance was set to *p* < 0.05.

## 3. Results

### 3.1. Bacterial Stains

All microorganisms were isolated from different departments of General University Hospital of Alejandroupolis (Table 1). Regarding the type of biological material from which the clinical strains were isolated, 46 bacterial strains (47%) were sourced from patient wounds, 38 from blood samples (39%), 5 strains from pus samples (5%), followed by 4 tissue samples (4%), 2 ocular discharge samples (2%), 1 strain isolated from a peritoneal fluid sample (1%), 1 strain from a graft sample (1%), and 1 strain from a bronchial secretion sample (1%).

### 3.2. Phenotypic Antimicrobial Resistance of Klebsiella pneumoniae

Of the 98 bacterial strains for which the determination of the antimicrobial resistance of bacterial strains (MICs) was performed using the Vitek 2 Systems automated method, 75 strains appeared resistant to the four classes of carbapenems (Imipenem, Meropenem, Doripenem, and Ertapenem) with values ≥16 R, ≥16 R, ≥8 R, and ≥8 R, respectively, for each carbapenem. In addition, 10 strains were shown to be resistant to the four classes of carbapenems (Imipenem, Meropenem, Doripenem, Ertapenem) with values of 8 R, ≥16 R, ≥8 R, ≥8 R, respectively, for each carbapenem.

Also, 6 strains appeared resistant to the four classes of carbapenems (Imipenem, Meropenem, Doripenem, and Ertapenem) with values ≥16 R, ≥16 R, ≥8 R, and 4 R, respectively, for each carbapenem. Additionally, 2 strains were shown to be sensitive to the four classes of carbapenems (Imipenem, Meropenem, Doripenem, and Ertapenem) with values ≤0.25 S, ≤0.25 S, ≤0.12 S, ≤0.5 S, respectively, for each carbapenem.

Furthermore, 1 strain appeared resistant with values 0.5 R, ≥16 R, 4 R, ≥8 R, and 1 strain appeared resistant with values ≤0.25 R, 1 R, 0.5 R, 4 R, 1 strain appeared resistant with values ≥16 R, 8 R, ≥8 R, ≥8 R, and 1 strain appeared as resistant with values of 4 R, ≥16 R, 4 R, 4 R and finally, 1 strain appeared as resistant with values of 8 R, ≥16 R, 4 R, and 4 R. The distribution of MICs/Cat values of the 4 carbapenems using the automated method Vitek 2 Systems is showed in Table 2.

In total, of the 98 bacterial strains studied, 96 appeared resistant to all classes of carbapenems (Imipenem, Meropenem, Doripenem, and Ertapenem), while only two strains (2.04%) appeared sensitive to carbapenems.

### 3.3. Detection of Carbapenemases Production Using Lateral Flow Immunoassay (LFIA)

Considering that the 96 strains have shown a resistant phenotype (R) out of a total of 98 strains when determining microbial resistance with the automated method Vitek 2 Systems, the immunochromatographic method (ICT) in terms of KPC enzymes detected 51 positive strains (53.12%) and 45 negative strains (46.87%). Regarding the immunochromatographic method (ICT) for OXA-48-like enzymes, 3 positive strains (3.12%) and 93 negative strains (96.87%) were detected. Regarding the two strains that appeared to have a susceptible phenotype (S) when determining antimicrobial resistance with the automated method Vitek 2 Systems to the four classes of carbapenems (Imipenem, Meropenem, Doripenem, and Ertapenem), no strain KPC or OXA-48 producer was detected using the immunochromatographic method (ICT).

Regarding metallo-β-lactamases VIM, NDM, and IMP, of the 96 strains that showed a resistant phenotype (R) out of a total of 98 strains, 40 (38.4%) produced carbapenemase. 32 (30.72%) strains showed the ability to produce NDM carbapenemases, and 8 (7.68%) VIM carbapenemase strains were also found. No strain was found capable of producing IMP-type carbapenemase. The two strains that appeared to have a susceptible phenotype (S), determined antimicrobial resistance with the automated method Vitek 2 Systems to the four classes of carbapenems (Imipenem, Meropenem, Doripenem, and Ertapenem). No strain showed the ability to produce VIM, NDM, or IMP type carbapenemases using the immunochromatographic method (ICT) (Table 3).

### 3.4. Multiplex Real-Time PCR for the Detection of KPC, OXA-48-like, VIM, NDM, and IMP Genes

As presented in Table 4, considering only the 96 strains that showed a resistant phenotype (R) out of a total of 98 strains when determining antimicrobial resistance with the automated method Vitek 2 Systems, the molecular method Multiplex Real-Time PCR in terms of KPC enzymes detected 86 positive strains (89.6%) and 10 negative strains (10.41%). Regarding the molecular method Multiplex Real-Time PCR for OXA-48 like enzymes, 14 positive strains (14.6%) and 82 negative strains (85.41%) were detected. Regarding the two strains that appeared to have a susceptible phenotype (S) when determining antimicrobial resistance with the automated method Vitek 2 Systems to the four classes of carbapenems (Imipenem, Meropenem, Doripenem, and Ertapenem), these were detected positive only in terms of genes KPC by the molecular method.

**Figure 1:**
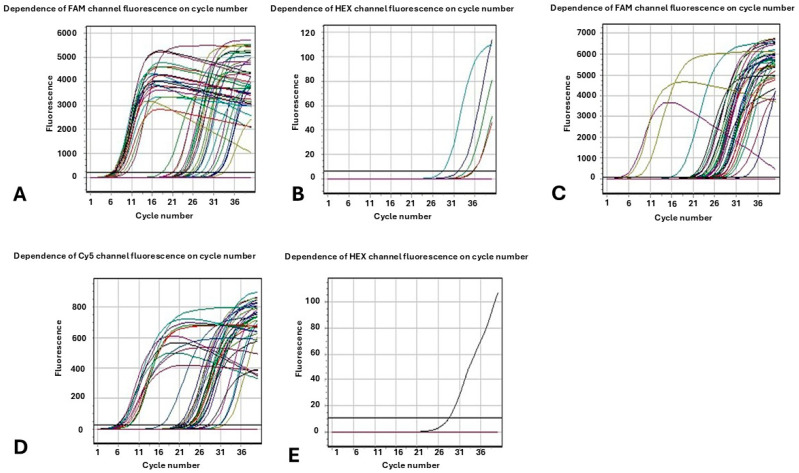
Fluorescence as a function of cycle number. The results of the Multiplex Real-Time PCR molecular method for the (**A**) KPC enzymes (channel FAM); (**B**) OXA-48 similar enzymes (channel HEX); (**C**) VIM enzymes (channel FAM); (**D**) NDM enzymes (channel CY5); (**E**) IMP enzymes (channel HEX).

Regarding metallo-β-lactamases VIM, NDM and IMP, of the 96 strains studied, 86.4% of the strains under study carry simultaneously two of the three carbapenemase genes NDM and VIM. A very small percentage, 4.8%, carries only the NDM gene. In 3.06% of the samples the VIM gene was detected, while the IMP gene was not found in any strain (Figure 1). Regarding the two strains that appeared to have a susceptible phenotype (S) when determining antimicrobial resistance with the automated method Vitek 2 Systems to the four classes of carbapenems (Imipenem, Meropenem, Doripenem, and Ertapenem) these were detected positive only in terms of genes VIM and NDM by the molecular method.

### 3.5. Comparative Analysis of Carbapenemase KPC and OXA-48 Production from Intensive Care Units (ICUs)

Considering the total of 22 strains originating from the Intensive Care Units (ICUs) using the immunochromatographic method regarding KPC enzymes, 11 positive strains (50%) and 11 negative strains (50%), were detected. Using the Multiplex Real-Time PCR method regarding KPC enzymes, 21 positive strains (95.5%) and 1 negative strain (4.5%) were detected revealing the significant presence of KPC producers among isolates originating from ICUs.

Furthermore, using the immunochromatographic method regarding OXA-48 enzymes, 2 positive strains (9.09%) and 20 negative strains (90.90%) were detected. Using the Multiplex Real-Time PCR method regarding OXA-48 enzymes, 3 positive strains (13.6%) and 19 negative strains (86.36%) were detected.

## 4. Discussion

*Kl. pneumoniae* play an important role in the global crisis of the emergence of resistant bacteria, with the species studied among others having the strongest impact on the development of antimicrobial resistance. Hospital-acquired infections due to carbapenem-resistant strains of *K. pneumoniae* demonstrate the highest degree of resistance, which is confirmed both by present research, and by a previously reported study [24].

In particular, during the study, three methods were used: two phenotypic approaches (measurement of microbial resistance using the Vitek 2 Systems automated system and lateral flow immunoassay-LFI) and one molecular detection method in order to test in vitro the potential of the microbial strains to produce carbapenemases enzymes, which play a central role in the phenomenon of microbial resistance against carbapenems.

Initially, regarding the detection of antimicrobial resistance using the automated system Vitek 2 Systems in total, of the 98 bacterial strains studied, 96 appeared resistant to all classes of carbapenems (Imipenem, Meropenem, Doripenem, and Ertapenem), while only two strains appeared sensitive to carbapenems. Automated testing of antimicrobial susceptibility, such as the Vitek 2 Systems, that were used in this study, is common in clinical microbiology laboratories, but they are not able to detect all resistant microorganisms. Leegaard TM et al., report that remarkable errors were identified for carbopenemes like ertapenem [25]. What should be noted is that, unexpectedly, the production of carbapenemases by enteric bacilli does not necessarily confer a significant degree of resistance to carbapenems. On the other hand, enterobacterial clinical strains in which no significant carbapenemase enzyme activity is found may exhibit high MICs to carbapenems [26].

Consequently, regarding the immunochromatographic method (ICT) in terms of KPC enzymes, of the 96 resistant strains according to the Vitek 2 automated method, 51 positive strains (53.12%) and 45 negative strains (46.87%) were detected, while regarding the molecular method of Multiplex Real-Time PCR for KPC enzymes from the 96 resistant strains, 86 positive strains (89.6%) and 10 negative strains (10.41%) were detected. Interestingly, we observe two groups for KPC during the PCR, but it may be due to parameters such as different expression of this gene on bacterial strains.

In relation to the immunochromatographic method (ICT) in terms of OXA-48 like enzymes, out of the 96 resistant strains with the Vitek 2 automated method, 3 positive strains (3.12%) and 93 negative strains (96.87%) were detected, while in terms of the molecular Multiplex Real-Time PCR method for OXA-48 like enzymes from the 96 resistant strains with the Vitek 2 automated method, 14 positive strains (14.6%) and 82 negative strains (85.41%) were detected. This result differs from previously published data by Kettani et al., who showed 100% agreement with PCR for the detection of carbapenemase OXA-48 [27].

Regarding the immunochromatographic method (ICT) in terms of VIM enzymes, of the 96 resistant strains according to the Vitek 2 automated method, 8 positive strains (7.68%) and 88 negative strains (92.32%) were detected, while regarding the molecular method Multiplex Real-Time PCR, the simultaneous detection of VIM and NDM enzymes from the 96 resistant strains, reached the 90 positive strains (86.4%) and 6 negative strains (13.6%), and the detection of VIM enzymes only was 3 positive strains (3.06%) and 93 negative strains (96.94%).

In relation to the immunochromatographic method (ICT) in terms of NDM enzymes, out of the 96 resistant strains with the Vitek 2 automated method, 32 positive strains (30.72%) and 64 negative strains (69.28%) were detected, while in terms of the molecular Multiplex Real-Time PCR method for NDM enzymes, only 5 positive strains (4.8%) and 91 negative strains (95.2%) were detected out of the 96 resistant strains with the Vitek 2 automated method.

Regarding the immunochromatographic method (ICT) and molecular method Multiplex Real-Time PCR in terms of IMP enzymes, no positive strain was detected with any of these methods.

These results led to the conclusion that the Multiplex Real-Time PCR revealed a higher percentage of positive strains for KPC, OXA-48 like, VIM, NDM, and IMP enzymes, which, using only the immunochromatographic method (ICT), would remain non-detectable.

Regarding the two strains that appeared to have a susceptible phenotype (S) when determining antimicrobial resistance with the Vitek 2 Systems automated method to the four classes of carbapenems (Imipenem, Meropenem, Doripenem, and Ertapenem), these were detected as positive only in terms of genes *bla*KPC, *bla*VIM, and *bla*NDM and only by the molecular method. Therefore, only the use of the immunochromatographic method (ICT) could not reveal the existence of these enzymes in these bacterial strains.

The use of the molecular method (Multiplex Real-Time PCR) in the field of detection of bacterial strains capable of producing carbapenemases has been applied in several studies [18] that utilize this method for this purpose and indeed with excellent sensitivity (100%), and specificity (100%) rates for the detection of an epidemic KPC *K. pneumoniae* ST258 clone.

In addition, another study [28] developed a multiplex real-time PCR method for the rapid detection of carbapenemase genes, which showed 100% agreement with the results of genotyping, which had preceded it. Finally, another study [29] by Zee van der A. et al. came to confirm the development of a Multiplex Real-Time PCR for the detection of cabapenemases OXA-48, VIM, IMP, NDM, and KPC with a sensitivity and specificity of 100%. Therefore, the results of these studies are consistent with the results of the present study, which demonstrated the importance of the molecular method for the identification of strains capable of producing carbapenemases, since the molecular detection method is considered the “gold standard” method for the detection of these enzymes [30].

Considering the immunochromatographic method (lateral flow immunoassay-LFI), as proposed by several studies among others [23], the sensitivity and specificity rates, which the present study demonstrated using the molecular method as the reference method for the enzymes KPC, OXA-48 like, VIM, NDM, and IMP are not in agreement with the results of a number of studies [19,20,21,30] which have demonstrated very high sensitivity and specificity rates of the multiplex immunochromatographic assay of 100% and 95.3% to 100%, respectively, for the detection of strains capable of producing different types of carbapenemases (OXA-48 like, KPC, NDM, IMP, and VIM type) supporting and urging the introduction of these methods into the diagnostic laboratory routine as a rapid, reliable and easy-to-use test for the detection of such bacterial strains.

Another point that would be particularly important to mention is the prevalence of KPC positive strains of the species compared to OXA-48 like positive strains in intensive care units (ICUs).

In more detail, from the total of 22 strains from the Intensive Care Units (ICU) using the immunochromatographic method regarding KPC enzymes, 11 positive strains (50%) and 11 negative strains (50%) were detected, while using the Multiplex Real-Time PCR method in terms of KPC enzymes, detected 21 positive strains (95.5%) and 1 negative strain (4.5%).

On the other hand, using the immunochromatographic method regarding OXA-48 enzymes, 2 positive strains (9.09%) and 20 negative strains (90.90%) were detected. Using the Multiplex Real-Time PCR method regarding OXA-48 enzymes, 3 positive strains (13.6%) and 19 negative strains (86.36%) were detected.

At this point it is necessary to note that the results of the present study agree with the results of other studies that were conducted in ICUs of tertiary care hospitals with KPC enzymes showing a significant prevalence of 74% (122 clinical strains) [31]. Nevertheless, it should be noted that recently a shift has been observed epidemiologically in terms of the detection of OXA-48 strains in areas of Greece.

## 5. Conclusions

Bacterial strains of the carbapenem-resistant species exhibiting the ability to produce carbapenemases are a particularly worrisome problem, especially in hospital settings. Therefore, the detection and the clear epidemiological picture, as well as the tracking of the dispersion pattern of such strains, are an imperative in the context of prevention and control measures to protect public health. Multiplex Real-Time PCR molecular methods are more sensitive than the immunochromatography method. Compared to the results obtained from the immunochromatography kit, we concluded that with the multiplex Real-Time PCR, a much larger percentage of the strains under study were revealed to carry class A, D, and B type carbapenemases. Even though they are expensive procedures that require specialized personnel, laboratory equipment, and know-how, molecular methods will remain as the reference methods (“gold standard”) in the field of detection of such strains. In addition, further studies investigating the sensitivity and specificity of LFIs should be carried out so that they can be exploited as a rapid, reliable, and easy-to-use tool in the field of carbapenemase epidemiology and prevention of the spread of carbapenemases to protect public health. In the future, this study could be carried out on a wider scale, including a larger number of bacterial isolates. Moreover, we could study the existence of resistance genes to other antibiotics besides carbapenems. It would also be important to investigate in vitro the molecular mechanisms that lead to the expression and transmission of such resistance genes, thus discovering potential targets to combat such infections.

## Figures and Tables

**Table 1:** Bacterial isolates by departments.

Departments	Number of Strains *n* (%)
Surgical	*32* (33%)
ICU	*22* (22%)
Internal medicine	*11* (11%)
Hematology	*7* (7%)
Neurology	*6* (6%)
Nephrology *	*4* (4%)
Cardiology **	*4* (4%)
Neonatology	*3* (3%)
Oncology	*2* (2%)
Orthopedic	*2* (2%)
Pulmonology	*2* (2%)
Urology	*2* (2%)
Special Infections Unit	*1* (1%)

ICU Intensive Care Unit; * Includes Nephrology department, Peritoneal Dialysis Unit, and Dialysis Unit; ** CCU Coronary Care Unit.

**Table 2:** Antimicrobial resistance of *Klebsiella pneumoniae* isolates with the automated method Vitek 2 Systems.

MICs	N *	Percentage (%)
IMI ≥ 16 R MER ≥ 16 R DOR > −8 R ETP ≥ 8 R	75	77
IMI ≥ 16 R MER ≥ 16 R DOR ≥8 R ETP 4 R	6	6
IMI 8 R MER ≥16 R DOR ≥8 R ETP ≥ 8 R	10	10
IMI 0.5 R MER ≥ 16 R DOR 4 R ETP ≥ 8 R	1	1
IMI ≤ 0.25 R MER 1 R DOR 0.5 R ETP 4 R	1	1
IMI ≥ 16 R MER 8 R DOR ≥ 8 R ETP ≥ 8 R	1	1
IMI 4 R MER ≥ 16 R DOR 4 R ETP 4 R	1	1
IMI 8 R MER ≥ 16 R DOR 4 R ETP 4 R	1	1
IMI ≤ 0.25 S MER ≤ 0.25 S DOR ≤ 0.125 ETP ≤ 0.5 S	2	2

* Total number of bacterial *Klebsiella pneumoniae* strains.

**Table 3:** Immunochromatographic method (ICT) for the detection of KPC, OXA-48 like, VIM, NDM, and IMP enzymes.

ICT	Positive	Negative
KPC	51 (53.12%)	45 (46.87%)
OXA-48 like	3 (3.12%)	93 (96.87%)
VIM	8 (7.68%)	88 (92.32%)
NDM	32 (30.72%)	64 (69.28%)
IMP	0	96 ^1^

^1^ The results were calculated to the total of 96 bacterial strains that presented a resistant phenotype according to automated method Vitek 2 Systems.

**Table 4:** Multiplex Real-Time PCR (ICT) for the detection of KPC, OXA-48 like, VIM, NDM, and IMP enzymes.

Multiplex Real-Time PCR	Positive	Negative
KPC	86 (89.6%)	10 (10.41%)
OXA-48 like	14 (14.6%)	82 (85.41%)
VIM+NDM	90 (86.4%)	6 (13.6%)
VIM	3 (3.06%)	93 (96.94%)
NDM	5 (4.8%)	91 (95.2%)
IMP	0	96 *

* The results were calculated to the total of 96 bacterial strains that presented a resistant phenotype according to automated method Vitek 2 Systems.

## Data Availability

The original contributions presented in this study are included in the article. Further inquiries can be directed to the corresponding author.

## Abbreviations

The following abbreviations are used in this manuscript:

PCR	Polymerase Chain Reaction
ICT	immunochromatographic method ICT
MICs	minimum inhibitory concentration
MDR	multidrug-resistant bacterium MDR

## References

[1] Gröndal H. (2018). The Emergence of Antimicrobial Resistance as a Public Matter of Concern: A Swedish History of a -Transformative Event. Sci. Context.

[2] Abraham E., Chain E. (1940). An Enzyme from Bacteria Able to Destroy Penicillin. Nature.

[3] Bud R. (2007). Penicillin: Triumph and Tragedy.

[4] Lee C.R., Lee J.H., Park K.S., Kim Y.B., Jeong B.C., Lee S.H. (2016). Global dissemination of carbapenemase-producing klebsiella pneumoniae: Epidemiology, genetic context, treatment options, and detection methods. Front. Microbiol..

[5] Won S.Y., Munoz-Price L.S., Lolans K., Hota B., Weinstein R.A., Hayden M.K., for the Centers for Disease Control and Prevention Epicenter Program (2011). Emergence and rapid regional spread of Klebsiella pneumoniae carbapenemase-producing Enterobacteriaceae. Clin. Infect. Dis..

[6] Podschun R., Ullmann U. (1998). *Klebsiella* spp. as nosocomial pathogens: Epidemiology, taxonomy, typing methods, and pathogenicity factors. Clin. Microbiol. Rev..

[7] Meletis G. (2016). Carbapenem resistance: Overview of the problem and future perspectives. Ther. Adv. Infect. Dis..

[8] Akajagbor D., Wilson S., Shere-Wolfe K., Dakum P., Charurat M., Gilliam B. (2013). Higher incidence of acute kidney injury with intravenous colistimethate sodium compared with polymyxin B in critically ill patients at a tertiary care medical center. Clin. Infect. Dis..

[9] Kitchel B., Rasheed J.K., Patel J.B., Srinivasan A., Navon-Venezia S., Carmeli Y., Brolund A., Giske C.G. (2009). Molecular epidemiology of KPC-producing *Klebsiella pneumoniae* isolates in the United States: 166 clonal expansion of multilocus sequence type 258. Antimicrob. Agents Chemother..

[10] Peirano G., Ahmed-Bentley J., Woodford N., Pitout J. (2011). New Delhi metallo-beta-lactamase from traveler returning to Canada. Emerg. Infect. Dis..

[11] Falagas M., Kastoris A., Kapaskelis A., Karageorgopoulos D. (2010). Fosfomycin for the treatment of multidrug-resistant, including extended spectrum beta-lactamase producing, Enterobacteriaceae infections: A systematic review. Lancet Infect. Dis..

[12] Sader H., Farrell D., Flamm R., Jones R. (2014). Variation in potency and spectrum of tigecycline activity against bacterial strains from U.S. medical centers since its approval for clinical use (2006 to 2012). Antimicrob. Agents Chemother..

[13] Morrill H., Pogue J., Kaye K., LaPlante K. (2015). Treatment options for carbapenem-resistant Enterobacteriaceae infections. Open Forum Infect. Dis..

[14] Giske C.G., Monnet D.L., Cars O., Carmeli Y., on behalf of ReAct-Action on Antibiotic Resistance (2008). Clinical and economic impact of common multidrug-resistant gram-negative bacilli. Antimicrob. Agents Chemother..

[15] Pitout J.D.D., Nordmann P., Poirel L. (2015). Carbapenemase-producing Klebsiella pneumoniae, a key pathogen set for global nosocomial dominance. Antimicrob. Agents Chemother..

[16] Martin R.M., Bachman M.A. (2018). Colonization, Infection, and the Accessory Genome of Klebsiella pneumoniae. Front. Cell. Infect. Microbiol..

[17] Bonomo R.A., Burd E.M., Conly J., Limbago B.M., Poirel L., Segre J.A., Westblade L.F. (2018). Carbapenemase-Producing Organisms: A Global Scourge. Clin. Infect. Dis..

[18] Chen L., Chavda K.D., Mediavilla J.R., Zhao Y., Fraimow H.S., Jenkins S.G., Levi M.H., Hong T., Rojtman A.D., Ginocchio C.C. (2012). Multiplex Real-Time PCR for Detection of an Epidemic KPC Producing Klebsiella pneumoniae ST258 Clone. Antimicrob. Agents Chemother..

[19] Sagıroglu P., Hasdemir U., Gelmez G.A., Aksu B., Karatuna O., Söyletir G. (2018). Performance of—RESIST-3 O.K.N. K-SeT‖ immunochromatographic assay for the detection of OXA-48 like, KPC, and NDM carbapenemases in Klebsiella pneumoniae in Turkey. Braz. J. Microbiol..

[20] Boutal H., Naas T., Devilliers K., Oueslati S., Dortet L., Bernabeu S., Simon S., Volland H. (2017). Development and Validation of a Lateral Flow Immunoassay for Rapid Detection of NDM-Producing Enterobacteriaceae. J. Clin. Microbiol..

[21] Song W., Park M.-J., Jeong S., Shin D.H., Kim J.-S., Kim H.S., Kim H.-S., Lee N., Hong J.S., Jeong S.H. (2020). Rapid Identification of OXA-48-like, KPC, NDM, and VIM Carbapenemase-Producing Enterobacteriaceae From Culture: Evaluation of the RESIST-4 O.K.N.V. Multiplex Lateral Flow Assay. Ann. Lab. Med..

[22] (2022). European Committee on Antimicrobial Susceptibility Testing, EUCAST Clinical Breakpoint Tables v. 12.0. https://www.eucast.org/fileadmin/src/media/PDFs/EUCAST_files/Breakpoint_tables/v_12.0_Breakpoint_Tables.pdf.

[23] Glupczynski Y., Evrard S., Ote I., Mertens P., Huang T.-D., Leclipteux T., Bogaerts P. (2016). Evaluation of two new commercial immunochromatographic assays for the rapid detection of OXA-48 and KPC carbapenemases from cultured bacteria. J. Antimicrob. Chemother..

[24] Karampatakis T., Antachopoulos C., Iosifidis E., Tsakris A., Roilides E. (2016). Molecular epidemiology of carbapenem-resistant *Klebsiella pneumoniae* in Greece. Future Microbiol..

[25] Leegaard T.M., Justesen U.S., Matuschek E., Giske C.G., on behalf of the NordicAST Study Group on Automated AST (2023). Performance of automated antimicrobial susceptibility testing for the detection of antimicrobial resistancein gram-negative bacteria: A NordicAST study. APMIS.

[26] Tzouvelekis L.S., Markogiannakis A., Psichogiou M., Tassios P.T., Daikos G.L. (2012). Carbapenemases in *Klebsiella pneumoniae* and Other *Enterobacteriaceae*: An Evolving Crisis of Global Dimensions. Clin. Microbiol. Rev..

[27] El Kettani A., Maaloum F., Nzoyikorera N., Khalis M., Katfy K., Belabbes H., Zerouali K. (2021). Evaluation of the Performances of the Rapid Test RESIST-5 O.O.K.N.V Used for the Detection of Carbapenemases-Producing Enterobacterales. Antibiotics.

[28] Monteiro J., Widen R.H., Pignatari A.C., Kubasek C., Silbert S. (2012). Rapid detection of carbapenemase genes by multiplex real-time PCR. J. Antimicrob. Chemother..

[29] Van der Zee A., van der Zee A., Roorda L., Bosman G., Fluit A.C., Hermans M., Smits P.H.M., van der Zanden A.G.M., Witt R.T., van Coppenraet L.E.S.B. (2014). Multi-centre evaluation of real-time multiplex PCR for detection of carbapenemase genes OXA-48, VIM, IMP, NDM and KPC. BMC Infect. Dis..

[30] Rösner S., Kamalanabhaiah S., Küsters U., Kolbert M., Pfennigwerth N., Mack D. (2019). Evaluation of a novel immunochromatographic lateral flow assay for rapid detection of OXA-48, NDM, KPC and VIM carbapenemases in multidrug-resistant Enterobacteriaceae. J. Med. Microbiol..

[31] Protonotariou E., Meletis G., Pilalas D., Mantzana P., Tychala A., Kotzamanidis C., Papadopoulou D., Papadopoulos T., Polemis M., Metallidis S. (2022). Polyclonal Endemicity of Carbapenemase-Producing Klebsiella pneumoniae in ICUs of a Greek Tertiary Care Hospital. Antibiotics.

